# Liberation Medicine in Authoritarian Contexts: The Brutality of U.S. Mass Incarceration

**DOI:** 10.1007/s11013-026-10004-5

**Published:** 2026-09-26

**Authors:** Philippe Bourgois

**Affiliations:** Department of Psychiatry, Center for Social Medicine, University of California Los Angeles (UCLA), Los Angeles, United States

## Abstract

This article engages Führer and Vorhölter’s (2025) reinvigoration of Nancy Scheper–Hughes’s utopian call for liberation medicine in their special issue of *Culture, Medicine, and Psychiatry*. They offer a practical reconceptualization of liberation medicine as “concrete utopias” in the “medical undercommons”. Inspired by Scheper–Hughes’s humble call for “good-enough” ethnographic engagement in her documentation of a successful solidarity movement of single-mother agricultural laborers in Northeastern Brazil, I highlight ethnographically a (fragile) concrete utopian initiative: a peer-support program for inmates with psychosis spectrum conditions who are swept into the Los Angeles County Jail system’s overcrowded mental health units on a daily basis by routinized law enforcement sweeps of homeless individuals. Developed by former gang members (without mental illness) facing capital punishment charges, this remarkable initiative of solidarity, redemption, and anti-racism through radical caregiving in a claustrophobic carceral environment is carving out a temporarily safe space free from abuse and violence. The larger facility remains gang-controlled and is overwhelmed by what colleagues and I have been calling “predatory accumulation” and “necrogovernance” processes (Hart et al., 2024, 2025). The inmates are powerless, caught within the “carceral mesh” of U.S. political-economic corporate and opportunistic special interests. Furthermore, the discretionary power of “hyperpunitive” U.S. judges allows them to routinely impose Life Without Parole (LWOP) sentences on redeemed inmates incarcerated as adolescents. Inspired by Scheper–Hughes’s practical political optimism coupled with her love for ethnography and political activism, I end with eight practical suggestions for specific state bureaucratic reforms to foment public transparency about the useless cruelty of mass incarceration and the global rise of authoritarianisms in our perilous moment in global history.

## Introduction

In the 1990s, Scheper–Hughes presciently called for “liberation medicine” as a mobilizing utopian concept in the making. Führer and Vorhölter’s special issue revitalizes the concept with their framing of health as a human right foundational to all civil/political economic rights. Liberation medicine and concrete utopias are a breath of fresh air in the global catastrophe of the second Trump/MAGA presidency. Regaining power in January 2025, Trump fast-forwarded his U.S. version of authoritarian kleptocracy and militarism, attacking science, bankrupting major universities, ravaging public health, eliminating foreign aid, and revving up racialized xenophobic nationalism. He slashed social budgets, engorged the bloated budgets of law enforcement, created military/paramilitary agencies, and continues to celebrate oligarchical and corporate greed—classic examples of “U.S. predatory accumulation” (Blazquez & Lamotte, 2024; Bourgois, 2018) and “necrogovernance” (Hart et al., 2024, 2025).^1^ As this special issue goes to press (Summer 2026), Trump is running rampant globally, normalizing imperial mass civilian bombardments and random high seas killings, and kidnappings or assassinations of leaders of oil-rich nations. The American people appear indifferent to the global (and local) necrogovernance their rogue president is wrecking on the planet in their name.

Führer and Vorhölter (2025) provocatively merge two concepts – “concrete utopia” (invoking Wilder, 2022), and the “medical undercommons” (invoking van der Waal, 2024). They reference independent socialist and non-dogmatic Black liberation scholars, notably among them, Franz Fanon (1963). “Concrete utopia” and the “medical undercommons” render Scheper–Hughes’s utopian vision of liberation medicine more visible and more pragmatically humble.

Combining feminism with political economy, Scheper–Hughes incisively documents in *Death Without Weeping: The Violence of Everyday Life in Brazil* the oppression and struggles of all the poor—including men (see “Rebel Body” in this special issue). She refused to sanitize the reality of suffering. This critique of suffering and violence resonates with Gramsci’s critiques of “false” and “contradictory consciousnesses” (1992: 323); and with critiques of justifications of “virtuous violence” (Scheper-Hughes & Bourgois, 2004: 5) that also draw on Pierre Bourdieu’s (2001) “epicentral” theory of symbolic violence (Wacquant & Akçaoğlu, 2017: 57) that legitimizes the power hierarchies across his other core concepts “habitus” and “fields” of power.

Inspired by Scheper–Hughes’s ethnographic political engagement, I will draw from my carceral fieldwork that documents a remarkably precarious and unlikely concrete utopia where one would least expect to find it: a medical, inmate-led, peer-support program in the Los Angeles (LA) County Jail system. It was carved out against all odds as a space for sociable caregiving for inmates with psychosis spectrum conditions by two charismatic inmates (Craigen Armstrong and Adrian Berumen). They do not have mental illness but face capital punishment charges. To my surprise, their initiative was supported by the Sheriff’s Department leadership, but not by their rank-and-file officers or even by the director of jail medical services. More logically from a social forces political economic perspective, the supportive program collides with the inertia of the larger political economic carceral mesh that quasi-sociopathically builds and maintains U.S. mass incarceration. Nevertheless, this spontaneously organic inmate-led peer-support program reduces some of the violent trauma inherent to incarceration. Most importantly it benefits two of the most oppressed populations in the USA: 1) vulnerable street-gang inmates convicted of murder; and 2) homeless inmates with psychosis-spectrum disorders—most of whom have dual diagnoses of substance use/schizoaffective disorders.

## Defining Liberation Medicine

Scheper–Hughes sketched her initial definition of liberation medicine in a three-page exploratory proposal under a header: “Toward a Liberation Medicine: A Pedagogy for Patients (and Practitioners).” She embedded it midway through her ethnographic masterpiece, *“Death Without Weeping: The Violence of Everyday Life in Brazil”* to call attention to the concept (Scheper-Hughes, 1992: 213–215). Surprisingly, 34 years have passed; yet, this special issue is the first time anthropologists (many with MDs) are elaborating the concept ethnographically and globally.

*Death Without Weeping* brought liberation medicine alive ethnographically for the first time. Scheper–Hughes did it by immersing herself in grassroots struggles for survival and dignity for survival and dignity among the single mothers of the Alto de Cruzeiro, a shantytown in the sugar plantation belt of northeastern Brazil. She also interviewed and sometimes confronted plantation executives, state bureaucrats and especially the doctors misdiagnosing the “invisible genocide” of infants from diarrheal disease (Scheper-Hughes & Bourgois, 2004: 5). The struggles of the Alto women, men, and children became her longest ethnographic site and one of her primary political commitments. She returned probably a dozen times to the steep muddy hillside throughout her lifetime.

Scheper–Hughes first took up residence in the Alto as a 20-year-old Peace Corps volunteer (1964–1966) working in public health/community development outreach (Scheper-Hughes, 1992: 5). Lack of food and potable water was malnourishing her neighbors and killing their most vulnerable infants. *Death Without Weeping* compassionately describes how shantytown mothers were compelled to triage their weakest–usually youngest–starving infants into accelerated deaths. They desperately wanted to cut short the suffering of the weakest to salvage what little food and energy might benefit stronger siblings with better chances of survival.

Scheper–Hughes and the shantytown mothers were swept into the alphabetizing and political consciousness-raising popular education movement of liberation theology, inspired by the Brazilian Paulo Freire’s (1970) pro-socialist ecumenical and atheist-inclusive interpretation of God’s “preferential option for the poor”. This utopian movement mobilized the poor throughout Latin America into humanitarian socialist movements from the 1960s through 1980s (Gutiérrez, 1973)—with pockets persisting globally today.

The mothers founded a communal daycare center to take turns nursing one another’s babies so others could do backbreaking agricultural day labor without leaving their infants crying from hunger, dying alone in their shanty-shacks. The mothers subsequently expanded their solidary vision by digging a well to distribute potable water to all their neighbors. Their seizing of class consciousness is a classic liberation medicine example of a “concrete utopia.”

Scheper–Hughes’s politics during the 1960s and early 1970s were also inspired by the U.S. Black Civil Rights in the deep South and by the Black Panthers in Oakland closer to her home in Berkeley. She also worked closely with the “Boston Mental Patients Liberation Front” and with San Francisco’s chapter of ACT UP in the 1980s (ACT UP Oral History Project, 2026a, 2026b). These social movements demanding health and social justice are all prime examples of liberation medicine manifesting in the “medical undercommons” consistent with all the articles in this special issue (Aragon Martin, 2025; Dasgupta with the Ankur Writers Collective, 2025; Mair, 2026; Pinto, 2025). Notably, these social movements for social justice often endure or are crushed because of repeated revanchist right-wing backlashes. Their visionary utopianism often prevails, however, moving the needle of history forward.

As a committed U.S. public intellectual, Scheper-Hughes engages society’s burning contradictions with scholarly rigor, empathy, and humility (see also Borofsky’s, 2017, 2019 calls for “public anthropology”). In the public sphere, she may be best known for co-founding Organs Watch with her colleague Lawrence Cohen to combat the global trafficking of human organs. In her scholarly writings and in court, she castigated for-profit predatory U.S. and international medical surgeons for betraying low-income organ donors. At the height of postmodernism’s critique of ethnography in elite U.S. anthropology departments during the 1980s and early 1990s, her love for ethnography and political engagement prompted what she called “good-enough ethnography” (1992: 28–30). She was invoking the pediatrician/psychoanalyst Donald Winnicott’s concept of the “good-enough mother”—attuned and attentive albeit inevitably humanly fallible in the context of the structural oppression of women as primary caregivers for children in an overwhelmingly masculinist world. Frustrated by U.S. anthropology’s political paralysis clothed in righteousness and excessively self-referential academic critique, she asserted with eloquence, humility, and hope: “Like every other master artisan (and I dare say that at our best we are this), we struggle to do the best we can with the limited resources we have at hand—our ability to listen and observe carefully, empathically, and compassionately” (Scheper-Hughes, 1992: 28). Commonsensically and inclusively, she accepts that all humans are “…necessarily flawed and biased” (Scheper-Hughes, 1992: 28) but also insists that we not be paralyzed by this fact of the human condition.

## Mass Incarceration of People with Psychosis Spectrum Conditions

In the spirit of Scheper–Hughes’s celebration of high-stakes ethnography of extreme suffering, her politicized artisan’s humility, and Führer and Vorhölter’s provocative highlighting of the power of the “concrete utopia” in the current terrifying moment of rising authoritarian regimes, I have edited a brief composite extract from fieldnotes that combine observations from three different LA County Jail tours spanning summer 2016 through winter 2018. First, I set the broader context of the political economic and ideological abandonment of people with psychosis spectrum conditions that has resulted in their hyperincarceration in jails across the USA.

Comprising eight mega-sized, custodial facilities, the LA Jail system is the largest municipal carceral gulag in the world (with the recent exception of El Salvador since 2024). On any given day, the “average daily inmate population” has ranged from overcrowded highs of 23,000 in 1998 (Wacquant, 2002) to still very crowded lows of the 12,000s during COVID in 2020 to the 13,000s at the present (Los Angeles County Sheriff’s Department, 2024, 2025a, 2025b). LA law enforcement (police, sheriffs, and highway patrol officers) arrest approximately 200,000 individuals annually (see remarkable statistical figures presented in Los Angeles County Sheriff’s Department (2024)). Most are released “on their own recognizance” within days or weeks. The LA Jail system is staffed by correctional officers and sheriffs and has a notorious history of ongoing class action lawsuits. These include numerous poorly monitored civil rights legal settlements since the 1970s that have not been implemented (U.S. Department of Justice, 2015). Most of the settlements focus on the systemic abuse of inmates with serious mental illness. Despite constituting less than six percent of the U.S. population, inmates with psychosis spectrum conditions constitute 50-60% of the total daily inmate population of the LA Jail system (National Institute of Mental Health, 2022; Los Angeles County Sheriff's Department, 2025a; for national prevalences, see James & Glaze, 2006; Bronson & Berzofsky, 2017).

Every day and night, Sheriff’s personnel jam new arrestees into holding cells where they undergo a chaotic “medical processing” triage in two basement floors of the Twin Towers facility, located along the underground tunnels connecting it to the more violent, gang-controlled Men’s Central Jail. Arrest can be life-threatening for individuals with co-occurring psychosis and alcohol and/or psychotropic drug use because of delirium tremens from alcohol withdrawal. These distressing symptoms are easily confused with or compounded by methamphetamine intoxication, and heroin withdrawal, and sometimes provoke or mask suicidality. Grossly overcrowded U.S. jails are architecturally and procedurally designed for punishment and control. This punitive infrastructure pressures even decent clinicians to focus on weeding out “‘hideouts’ and ‘malingerers’” instead of caregiving.^2^

Inmates in the most florid states of psychotic distress are confined in the High Observation Housing (HOH)  cells of Twin Towers Jail. Most have diagnoses of schizophrenia-spectrum disorders. They are at risk of catastrophic bullying and sexual violence from both “general population” inmates (without mental illness) who fill the rest of LA County’s Jail facilities and also from sheriffs or correctional officers. These HOH mental health units are classified as “mandatory double occupancy” despite being quasi-solitary confinement-sized cells only about 1.8 x 2.7 meters (Los Angeles County Sheriff Civilian Oversight Commission, 2023). They barely have space for a narrow bunkbed and a seatless toilet bowl cemented into the floor. Sixteen of these claustrophobic cells are divided into “pods,” arranged arena style semi-circle along two split-level tiers around a small “common space” (about 8 x 13 meters).

Each floor of Twin Towers has a single panopticon-style (Bentham, 1995 [1787] Foucault, 1977) hexagon-shaped guards’ monitoring station with a bird’s-eye, 365-degree view into their floor’s six pods. Additional correctional officers rove nonstop scanning barcodes every 15 min throughout the pods of HOH cells and every 30 min for Medium Observation Housing pods, which are overcrowded dormitory cells about the size of a small elementary school gym. This barcode “safety monitoring” is merely one example of the dozens of mandates imposed by class action lawsuits responding to astronomical suicide rates in jail mental health units. The Men’s Central Jail facility is windowless—except for the administration offices on the top floors. It is the oldest, most overcrowded facility of the Los Angeles gulag. Inmates are squeezed into two rows of bunk beds lining both side walls of their cell. The so-called “windows” of Twin Towers are functionless six-inch-wide slits of reinforced glass admitting no natural light or air.^3^

## Composite Fieldnote (Winter 2016–Winter 2018)

Our carceral tour for Psychiatry residents traverses a long basement corridor from Men’s Central Jail. As we enter Twin Towers, we find ourselves tiptoeing through sewage pools from a stopped-up toilet-protest. An inmate is wailing in distress in his pod’s common room. He is shackled to one of several solid steel “spider tables” cemented into the floor. Three or four of these terrifying-looking structures occupy most of the “common spaces” of HOH pods. They have nodules welded onto each of their attached stools with chains dangling like menacing octopus tentacles. Corporate carceral furniture purveyors highlight this nodule/chain arrangement as a “customizable option.” Its classic neoliberal predatory corporate design maximizes the number of inmates who can efficiently be simultaneously handcuffed together, requiring only one guard.

To my horror, and presumably that of the psychiatry residents, we obediently, silently, uselessly pass single file by the chained inmate. The poor man is yanking at his manacled wrist as he attempts to walk around the table. He stumbles repeatedly, the ungainly, thick suicide smock tangling with his shackles. He is emaciated. It is unbearable to see! He starts shaking out his legs and yells loudly as we start exiting. Constricted by his shackles his rebellious feet splash into the sewage puddle pooling under his table. I suspect he is suffering from “restless leg syndrome” because several psychosis medications cause that distressing side effect. Egregiously, shackling to a spider table counts toward California’s weekly mandate of minimal out-of-cell “free time” that contradictorily is supposed to include three hours of “exercise” and seven hours of “recreation.”

The two pods staffed by the inmates, Craigen Armstrong and Adrian Berumen, who both face capital punishment charges, are a total contrast to the rest of Twin Towers. Their pods are dry, calm, quiet, and clean-smelling. These two inmates are developing a pilot peer-support program for inmates with serious mental illness. They serve fewer than 60 inmates out of the 5000–6500 prisoners who urgently need equivalent care on any given day inside the LA Jail system (Los Angeles County Sheriff’s Department, 2022, 2024, 2025a). Almost surreally, their common spaces are adorned with colorfully hand-painted motivational posters exuding a quasi-cheery kindergarten welcome. Jarringly, however, spider chain tables also fill their common spaces.

The attending Correctional Health psychiatrist who is leading our tour, “Dr. X” ushers us into the guardroom and cautiously and politely requests permission from the correctional officers clustered in the officer’s central monitoring station for our tour group of UCLA psychiatrists to “talk to the peer-support inmates.” We stand obediently nervously and silently waiting for a response. The guards are mostly expressionless, bored, or daydreaming. A few are streaming Hollywood action movies or pornography on their security monitors. Occasionally a guard smiles or laughs at a feed on a security monitor. None show interest in interacting with us. Embarrassed, the attending psychiatrist re-clears her throat to prompt a reply. The slouching officer closest to us sits up and smiles a quick assent. (I am always surprised to see highly skilled medical doctors in U.S. carceral facilities subordinate themselves to guards, squelching the hierarchical professional habitus instilled in them during medical school.)

Relieved, our psychiatrist guide eagerly waves over to Craigen and Adrian, the two inmate peer-support providers. Dr. X has been trying for several years to improve psychiatric care for jailed inmates. She is volunteering (pro-bono) to supervise UCLA residents to do a rotation in the jail. Adrian enters the hermetic bubble of the central guard’s monitoring station first. He is Latino and barely looks 18. Like a deer caught in headlights, he forces a wide smile at the intimidatingly formally dressed visitors and bored correctional officers. He asks shyly: “Do you want us to do …like a presentation?” Awkwardly we fail to answer, still too disoriented by what we saw in Men’s Central Jail—especially the infrastructural cruelty of the spider table shackling we passed upon entering this facility.

Craigen joins us, while I am nervously over-cheerily attempting to break the awkward ice. I introduce the psychiatrists-in-training, explaining that they are, possibly, interested in working inside Twin Towers and that it would be helpful for them to hear about “the remarkable program you and Adrian have been developing.” Craigen is Black and about twice Adrian’s age. In a calming, laconic voice he describes the program’s history: “We were selected as trustees [unpaid inmate workers] and told to be Merit Masters [inmates modeling good behavior]. But they also asked us to teach court competency.”

Craigen explains with urgency the tragically low bar of court competency:

“We reassure the guys that they can get out of here, but first they need to go to court and identify who is the judge, the lawyer, and the prosecutor. [Sadness tingeing his voice] But right away we saw they needed much more help. Almost everyone here is homeless and most smoke crystal [methamphetamine the drug-of choice in LA’s homeless Skid Row]. They often don’t remember what they did or know why they’re here.

[More cheerily] So we start by telling them they’re not incompetent in life—only in court. We tell them where the judge, the defense attorney and the prosecutor will be sitting… And to say, ‘No contest, your honor,’ to the judge. [Adrian interrupts enthusiastically] We have… a little curriculum [hesitating on the cacophonous academic word] Matter of fact, please! We need your help with it.”

One of the psychiatric residents asks what training they received. Craigen smiles gently, shaking his head, “Nahhh … We didn’t really know about mental illness. I mean … we seen homeless people on the streets out there, but … we just thought they were … like … bums. We never really thought nothing’ about it.”

As if intuitively echoing Scheper–Hughes’s 1990s good-enough manifesto, Craigen summarizes their pedagogical approach:

“We began by talking to them and listening to their stories… Finding out where they live; what they like… What they need.

It’s really not all that hard. We’re here 24/7, just like them, locked in our cells every night. When they wake up every morning, they see us in the common room already cleaning up. They call us over… We talk to everyone. They trust us now. All of them.”

Craigen and Adrian take turns describing their “collective activities” of “daily skills and self-care”: “We do a Monday Double Scrub, in the common room all together.” But first we had to start by showing them you cannot step right where you mop.”

They praise the officer (Sergeant Flores) supervising Craigen’s pod, who is exceptionally supportive. After a few months, he persuaded Food Services to allocate a coffee machine to the peer-support pods. This enabled them to start their “Red Star Reward program,” a “reciprocity-based approach,” to promote inclusive pro-sociality:

It’s really about teaching them how to socialize … to understand you gotta give something in order to get something. When they came in, they really didn’t know how to help themselves. Most been on their own too long in the streets….

At first we gave coffee for participation or good behavior. But now everyone participates—even the ones who used to smear feces and self-harm. So we just give everyone coffee whenever they ask for it.

Beaming now, Adrian adds enthusiastically:

See, all they really need is for someone to show them a little love. That’s why we started wake-up exercises: Craigen and I start the day at 5am and do exercises together first. We saw them imitating us from inside their cells. So now we do it all together every morning—even during lockdowns.

The weekends are hard on the guys: some decompensate. 48-hour lockdowns are just too long for them. But the exercises and extra cups of coffee help a lot on those long weekends.

A resident asks admiringly, “How do you motivate ‘self-grooming’?” Adrian chuckles at the memory:

See, it’s all about the females. [innocently blushing and shrugging when he notices his term “the females” startles several of us]. Oh! You gotta understand, that’s all you have to work with here. So we tell them, ‘You might want to get next to a female someday when you free again. But you gotta brush your teeth to do that.

Matter of fact…‘Trooper! [laughing to reassure us that the inmates recognize he is jesting] Your breath stinks! If you wanna keep talking to me, you gotta go brush your teeth right now!’

Craigen nods, adding empathetically:

But we see their gums started bleeding, because if you brush your teeth the wrong way, you hurt yourself. That’s when we realized we really didn’t do it right neither. [Smiling modestly] It’s complicated! So we requested a health tech cosmetologist and she taught all of us how you supposed to brush your teeth.

Pointing to the clock, Craigen signals that our visit has to end but gracefully adds a welcoming encouragement for the residents to return and train here.

Sorry, it’s time for us to prepare their food right now. The guys are hungry. But please come back! And next time you won’t see no spider tables in our pods no more… Just regular tables and chairs… No chains! … No shackles… Never!

## Interstitial Bureaucratic Procedural Reform in Carceral Context

Later, I learned that Craigen and Adrian’s supervisor, Sergeant Flores, impressed by the positive toothbrushing experience, requested that they be upgraded from “Trustee/Merit Masters” to a higher new status: “Mental Health Assistant (MHA).” They were allocated all-white button-down-shirt uniforms to signal bureaucratic Sheriff leadership level recognition of their expertise in the jail system’s unpaid inmate labor hierarchy. Most transformatively, Flores also submitted a work order to remove spider tables from all MHA pods.

Whether free or behind bars, most inmates have never before had positive contact with mental health services or any state institution. However, in the MHA pods, the subcontracted forensic psychiatrists ceased rushing perfunctorily through their caseloads. When rounding, they consulted first with Craigen and Adrian for patient updates and became willing to adjust medications—even after hours!—when sent notifications about urgent negative side effects from the MHAs through the intermediary of a sympathetic Health Tech (Sarah Tong) assigned to the unit. An early internal Corrections Health study also documented a spectacular sixfold decrease in suicide rates in the MHA units (Leyva, 2021: 29).

Adrian’s hostile deputy sheriff supervisor, in contrast to Craigen’s Sergeant Flores continued wantonly disrupting the calm Adrian had established and precipitated florid distress by cutting short inmate-patients’ “free time” in the common space. On my solo visits, Adrian’s supervisor would abruptly order me to leave “for security.” All one can do behind bars to maintain one's visiting rights is always immediately obey any custodial staff's orders or one may instantly lose access to entering LA Jail system facilities.

Shortly after the residents’ group tour, Sergeant Flores was promoted to Lieutenant and sent upstairs to an administrative office. The deputy sheriff assigned to replace Flores was hostile to the MHA program. Luckily his supervisor supported the program and reassigned that problematic deputy elsewhere. Most importantly Bruce Chase, the Head Assistant Sheriff of Custody Operations [aka ‘warden’], announced plans to expand the MHA program to all HOH pods throughout the entire jail system. Expansion has been slow, however, because of rank-and-file and low-level administrative ideological antagonism to rewarding any inmates.

Chase approved Flores’s crucial infrastructural renovation work order, and it remained an open work order that continues to enable spider table removal from newly established MHA-served pods. The MHA peer-support program painstakingly expanded from its initial two “pilot” pods in 2021 serving less than 60 inmates at a time to 23 pods by Spring 2026 serving over 500. It could easily triple in size to meet the “unmet need” for mental healthcare throughout the jail system. A satellite “pilot peer-support program” was established in the women’s jail at the end of the COVID pandemic. Typically, 60 to 70% of women prisoners have psychosis spectrum conditions. Chase participated in multiple high-profile social media and press venues with Craigen and Adrian), academics, and mental health advocates (including a podcast panel with MD/PhD Jeremy Levenson, mental health advocate/Sheriff’s Religious Volunteer Kerry Morrison, and me). Crucially, Chase explicitly asserted his belief in rewarding inmate redemption at public social media events (Morrison, 2021).

The program also solved the entry-level labor crisis for staffing jail mental health units. The few progressive and the larger number of lazy rookie Deputy Sheriffs working in Twin Towers now vie for assignment to MHA pods because there is no longer frequent need for violent “cell extractions” and there are virtually none of the routinized manifestations of extreme distress that generate self-harm in most mental health pods not served by the MHAs (e.g., head bashing, mutilation of wrist arteries, self-hanging, and feces–eating/smearing/hoarding that I witnessed while shadowing clinicians throughout the LA Jail system).^4^ Even the classic claustrophobic embodied protests (like toilet flooding or persistent wailing/crying) no longer occur on MHA pods. When inmates begin to express distress, they are rapidly comforted through sociable reassurance. It is a dramatic contrast from the large number of mental health pods that remain unserved by the MHA program. These extreme expressions of suffering have erupted in all of the over one dozen mental health units I have visited on visits inside U.S. and international carceral facilities over the past two decades.

A guilt-inflected PTSD plagues some of the bullies who abound in the labor forces of frontline U.S. law enforcement, carceral facilities, and military personnel. The minority who are openly empathetic often bear a sense of “moral injury.” For the past 2 years (2024–2026), there has been little need for guards in MHA units anymore. When I visit with photocopied medical texts or social science readings, I often have to wander throughout the floor searching for an officer to inspect each page of the photocopies before being allowed to give the readings to an MHA.

Most subsequent Sheriffs of Custody Operations have supported the program—notably Brendan Corbett and Robert Luna. Despite ostensibly impressive leadership support, hostility toward “coddling crazies” still pervades the older, mid-level rank-and-file deputy sheriffs, who adhere to the principle of maximum distrust and punitive control over inmates and visitors. This punitive hegemony has stymied expansion and may result in the ultimate implosion of the concrete utopia of the MHA program ultimate implosion beneath the bureaucratic routinized inertia of U.S. carceral hyperpunitivity. Antagonism is most vocally expressed among frontline police officers, best summed up by an officer’s flippant quip of “Crime over crazy” to an ethnographic collaborator, Blake Erickson (former MD/PhD student, now anthropologist/psychiatrist at Columbia).^5^

## Carceral Predatory Profiteering and Punitive Priorities within the Carceral Mesh

The ideological hegemony of maximum punitive control synchronizes with the predatory financial interests of the immensely costly public and private sector edifice that builds, manages, fills and maintains the U.S. carceral mesh. As a result since the 1980s, California’s judges and prosecutors (aka district attorneys) have been empowered by a constantly revised penal code with (as of 2026) approximately 33 “special circumstances”. They permit discretionary sentencing “length enhancements” by judges. This also encourages district attorneys to add overlapping or redundant criminal charges. In synchrony front-line law enforcement officers can arbitrarily arrest unruly, distressed, or merely homeless individuals. District attorneys have escalated their  flamboyantly bullying courtroom theatrics. These judicial calisthenics are crucial to the inertia of the multibillion-dollar carceral budget. Funding exploded when the formerly relatively robust educational and rehabilitative programs of the 1970s were suddenly dismantled in U.S. carceral facilities (Simon, 2014). The size of the carceral population rose rapidly in the mid-1980s and then skyrocketed in the 1990s. Shockingly, U.S. mass incarceration rose fastest when crime indexes—especially violent ones—started dropping rapidly in the 1990s. California led that carceral tsunami. The immense LA Jail system exploded from ~9000 inmates in 1980 to ~23,000 by 1998 (Wacquant, 2002). Crime dropped even lower over the next two decades, currently hovering at historic lows, without significantly decreasing the incarcerated population (Tapp & Coen, 2025).

The U.S. judicial system is notoriously slow, severe, and inconsistent. U.S. judges impose longer sentences than do their judicial peers in other nations. California’s populist “special elections” exacerbate hyperpunitivity. Most notable is the 1994 “Three Strikes Law” (invoking populist baseball lingo). Reconfirmed by voters as Proposition 184, it reaffirms the ability of judges to convert both long and short fixed sentences into LWOP (Life Without Parole) sentences. Judges can also “stack” multiple sequential “special circumstances enhancements” compounding the length of overlapping charges without jury trial oversight.  They can “trigger a strike” simply for two subsequent minor charges if a defendant has at least one previous felony. “Third strikes” condemn defendants to death from old age in prison. A “preliminary assessment” by the California Legislative Analyst’s Office of the Three Strikes law found that, “during the first eight months of implementation about 70% of all second and third strikes are for nonviolent and nonserious offenses” (Esparza et. al., 1995: 8; Vitiello, 1997). U.S. criminologists consistently document that wealthier and whiter criminals are sentenced more leniently. They often receive inconsequential civil court fines despite harming many hundreds or thousands of victims through white-collar racketeering schemes In contrast low-income, often racialized defendants in criminal courts generally receive lengthy carceral sentences for harming one individual (Gottschalk, 2015).^6^

Hyperpunitive judges discretionarily sentenced both Craigen and Adrian to life sentences despite the defendants' perfect record of redemptive service to society as incarcerated MHAs. Adrian and Craigen had over a dozen letters of support and multiple in-court testimonies from custody health personnel, academics, law enforcement officials, and U.S. federal Department of Justice personnel. With a flick of their gavels, two reactionary U.S. criminal court judges (Ronald Coen and Laura Laesecke) have propelled the entire MHA program into risk of collapse. Their chances of productive lives as freed redeemed former gangsters are devastated, and their family members are immersed in even more prolonged grief. The court proceedings dragged on uselessly for crimes committed (respectively) over one and over two decades ago.

Adrian fared only slightly better than Craigen in court. A juvenile district attorney offered Adrian a “fixed, determinate fixed plea deal” of “25 years” that by definition waived Adrian’s right to a jury trial and required Adrian to make a good-faith admission of guilt in the court record. Moments later Judge Laesecke, in hyperpunitive bad faith, contravened the intent of the district attorney’s plea deal by declaring an explicitly indeterminate potential sentence of “25 Years *to Life*”. In other words, Adrian now lives in prison under a Sword of Damocles of de facto LWOP: Any correctional officer in a bad mood can file a disciplinary “add-on charge” for a trivial or inadvertent infraction, which can legally transform Adrian’s 25-to-life sentence to a permanent life sentence without the right to a future parole hearing for good behavior or to a jury trial.

In comparable countries with more functional court systems, Laesecke might have been dismissed or at least reprimanded for flagrantly bullying defendants from her pulpit. In Southern California’s criminal court, however, she has performative license to bait terrified defendants as much as she likes. Adrian’s preliminary hearings coincided with Halloween week at the height of the COVID pandemic, and Laesecke wore a grinning wicked witch mask instead of the court-mandated medical COVID mask in the pulpit. Furthermore, her final indeterminate sentence was especially draconian because Adrian was only 17 at the time of his crime. In fact, Adrian’s juvenile co-defendant was freed from prison several years before Adrian was sentenced. He had waited nine years in jail for his few days in court.

Craigen fared even worse than Adrian. A Southern California Superior Court judge, Ronald Coen, railroaded him into LWOP, despite the fact that he too had been only 17 when he committed his first criminal “strike”; he was tried as an adult because of “special circumstances.” From the pulpit, Coen ridiculed the dozens of character witnesses testifying on Craigen’s behalf. In his sentencing preamble, he gloated visibly: “You [witnesses and family members] all come asking for mercy today. Well, I am the wrong man for that….” In 2009, the Southern California regional press nicknamed Judge Coen “Maximum Ron” for condemning 32 Californians to the death penalty (Friedman, 2009). No estimate is possible for how many defendants Coen has sentenced to LWOP because the United States does not oblige law enforcement or the judiciary to collect statistics.

Seventy-eight-year-old Maximum Ron’s reactionary superannuation (similar to that of half of the six most conservative U.S. Supreme Court judges) demonstrates in 2026 the urgency of mandatory judicial retirement ages and the need for public records transparency. Coen contributed actively to California becoming a historic leader of U.S. mass incarceration. In addition to press reports, the limited existing judicial archives document the ways he streamlined selection of juries for his death penalty trials to facilitate speedy jury condemnations and “stacked” multiple overlapping sentences for minor crimes running them “sequentially” (instead of “concurrently”). This allows him to legally impose LWOP without jury review (Anon, 2018; Friedman, 2009; Leon, 2015).

## Scholarly Humility, Utopianism, and Liberation Medicine in the Carceral Undercommons and the Challenge of Authoritarianism

In the face of overwhelming state carceral oppression and hopeless alienation, Armstrong and Berumen’s MHA program inspiringly violates the racist divide-and-conquer taboo imposed by prison gangs (and by most U.S. prison guards). Zero tolerance for interracial cooperation or friendship is enforced violently by prison gangs. Racism consolidates control over mutually alienated prisoners. U.S. hyperincarceration of gang members enables incarcerated gang leaders to collect “rent” because most street-based gang members get arrested chronically. The LA Jail system professionalized former adolescent street-corner gangs, driving (along with the firearms industry) the tragically high U.S. Black and Brown adolescent murder rates (Contreras, 2024). Risking their own lives from prison gang retaliation, Craigen and Adrian oblige new Mental Health Assistants in training to share cells with a prisoner from a different racialized ethnicity.

California’s ongoing judicial repression, however, demonstrates the precarity of utopian initiatives in authoritarian carceral bureaucracies. The contemporary Trump/MAGA regime further demonstrates the danger that authoritarianism will become institutionalized into a U.S. kleptocratic version of electoral fascism. Reinvoking Scheper–Hughes’s call for anthropologists to engage in “good-enough ethnography” by participating in “concrete utopias” prevents academic political paralysis. Unfortunately, concrete utopias remain effectively powerless in the face of the structural bureaucratic and political economic forces that maintain the U.S. carceral mesh. Corporate neoliberal greed thrives from its maintenance and expansion. Nevertheless, an immediate “good-enough” (practical short-term) means of decreasing some suffering within the U.S. carceral mesh is for skilled or entry-level healthcare providers, educators, researchers, social workers, etc., to obtain employment or to volunteer inside carceral facilities. It is hard to get inside carceral facilities legally, but when you do, you become a frontline witness to institutionalized state cruelty while possibly relieving, or at a minimum expressing solidarity in the face of the suffering of at least a few of society’s pariahs.

Inspiringly, concrete utopias pop up throughout the bloated U.S. carceral system. Prisoners enduring LWOP regularly muster the fortitude to educate themselves and seek atonement serving their peers—most frequently as jailhouse lawyers who “keep hope alive” (Kilgannon, 2026). Thirst for social justice and utopian community solidarity is as fundamental a human reflex as violence and self-interest. The MHA example is just another spontaneous liberation medicine initiative precariously remediating suffering in the carceral mesh’s “medical undercommons”. The contemporary crisis of increasingly for-profit neoliberal healthcare globally raises the stakes of utopianism. Utopian movements including liberation theology have always confronted militarism and fascism head-on.^7^

The USA urgently needs to expand its liberation medicine analytical repertoire to identify and denounce the entrenched mesh of predatory beneficiaries maintaining U.S. mass incarceration. Prisons facilitate corporate profiteering and spawn sprawling special interest constituencies. The mesh sucks in many diverse sectors: architects, construction and maintenance companies, conservative politicians, lobbyists, private equity investors, telecom monopolies, security systems, purveyors of protective gear, security video technology, institutional food, furniture with customizable nodules and dangling chains, and the bloated labor forces of law enforcement agencies, forensic health and social service providers and academic researchers. They—or rather we—all become entangled and thrive in the overfunded swamp of the extractive carceral mesh that urgently needs to be made transparent and dismantled.

Craigen and Adrian are Californian reincarnations of Gramsci’s “organic” public intellectuals (1992: 1). Their organic intellectual genius exudes from their handwritten books and pamphlets.^8^ Despite their draconian sentences, both men will continue to work for redemption and care for others inside their respective prisons. Virtually daily, U.S. judges squander the tremendous leadership potential and service to society of an untold number of carceral self-taught public organic intellectuals like them. The U.S. judicial system perpetuates a useless cycle of grief on the family members of the perpetrators and stirs up the pain of family members of victims and permeates low-income narcotics market-impacted communities (see Hart et al., 2025).

## Conclusion: Good-Enough Interstitial Public Intellectual Engagement in Authoritarian Contexts

In a debate with Noam Chomsky over public intellectual action, Foucault argued for the utility of “specific intellectuals” who become technical experts on or within the arcane interstices of state bureaucracies (Chomsky & Foucault, 1971). As specific intellectuals, we can practically engage in on-the-ground debates and promote larger institutional/political reforms while participating in humanitarian social movements (Bourgois & Schonberg, 2009: 297–320; and Messac et al., 2013: 180–181, 184–185; see also in southern and Baha California, Martinez, 2025; Levenson & Samra, 2025). For these reasons, I force myself to conclude with a preliminary list of eight only “good-enough” specific bureaucratic reforms. They might help identify the tentacles of the carceral mesh and render its predatory constituencies more accountable. My hope is that transparency over the state’s most repressive bureaucracies and corporate or kleptocratic opportunists will call attention to the grief that U.S. mass incarceration imposes on too many incarcerated people and their kin--especially mothers, children, and spouses. The eighth and final point may be too utopian to count as good-enough:

1. Increased access to visiting carceral facilities by family members, researchers, medical practitioners, and volunteers of any humane or useful kinds.
2. Mandatory public transparency of carceral population statistics including lawsuits, death rates/suicide attempts, and medical care.
3. Mandatory public transparency of carceral budgets and payments to all subcontractors profiting from maintaining/expanding carceral facilities including ICE concentration camps.
4. Regular inspections of all U.S. carceral facilities by international and national good-governance agencies.
5. Comparable severity of sentencing between criminal and civil courts so that corporate financial criminals stop receiving inconsequential fines while low-income felons (and sometimes only misdemeanants) stew for life behind bars.
6. Increased options for alternatives to incarceration within the carceral mesh from arrest to prison through preventative diversion into services, education, treatment, and rehabilitation rather than incarceration that lowers rather raises the cultural capital of perpetrators to reduce the power of organized crime networks that thrive inside U.S. carceral facilities.
7. Mandatory retirement ages for judges and systemic public transparency of their sentencing records to identify, and at least shame, hyperpunitive outliers.
8. Lobby the International Criminal Court in the Netherlands to indict and prosecute Trump for his war crimes and other crimes against humanity (bombardment of Iran, funding and arming the ethnic cleansing of Lebanon and Gaza, terrorization of millions of hardworking, honest undocumented immigrants, and starvation of countless more below subsistence-level families in low-income countries who formerly received remittances and the premature death of those who lost access overnight to medication and food when Trump arbitrarily slashed foreign aid.)

Transparency is simply a baby step toward alerting the public and promoting accountability for human rights violations and infrastructural carceral violence. The corporations and special interest constituencies that profit from the useless suffering of ongoing U.S. mass incarceration are the most intractable problem. To have any hope of addressing this problem, a social movement is needed. Given U.S. populist indifference in the age of predatory AI and kelptocratic right-wing revanchism, the U.S. carceral mesh will continue to expand and waste billions more public tax dollars, uselessly promoting global warming and ruining lives. Opportunistic politicians thrive by spawning populist moral panics over crime and insecurity (irrespective of whether crime indexes rise or fall). Militarism, authoritarianism, kleptocratic opportunism, and neoliberal policies that immiserate the working class and the unemployed are the bulwark of the carceral mesh. Concrete utopias and liberation medicine in the carceral mesh commons remind us that the U.S. carceral gulag is a project we can actively refuse. If Trump/MAGA’s klepto-authoritarian militarist mayhem prevails in the USA, an international boycott might wake up my fellow Americans.

## Data Availability

The article is a combination of literature review and presentation of a composite ethnographic fieldnote and additional ethnographic analyses  (edited for brevity and to protect confidentiality of respondents). It also analyzes scholarly and grey/archival online literature and public data sets  documenting U.S mass incarceration in the U.S. and Los Angeles.

## Footnotes

1 “Necrogovernance” emerges ethnographically from the perspective of those dying from exploitation and state repression inspired by Achilles Mbembe’s concept of “necropolitics” (2019) as does “predatory accumulation” from Marx’s “originary accumulation” (mistranslated into French and English as “so-called primitive accumulation”) (Marx, 1990 [1867]: 873-926).

2 For three especially insightful ethnographies of the Los Angeles County Jail, see Levenson (2024) on the contradictions of forensic psychiatry, Contreras (2024) on carceral gang-control and extreme violence, and Wacquant (2002) on the predatory cost of claustrophobic carceral infrastructure that propels U.S. mass incarceration. For a multi-century history of the racialization of LA County jails from 1771 to 1965 see Lytle Hernández (2017).

3 The greed of the predatory architectural and construction Planning corporation (HOK) Hellmuth, Obata, and Kassebaum (https://www.hok.com/), that built Twin Towers resulted in the County of Los Angeles suing the corporation for its overpriced flawed design before the first inmates were confined to the claustrophobic facility (Los Angeles Times, 2000).

4 On doctors and routinized violence against inmates in jail by doctor anthropologists see the vivid ethnographic accounts in Culture, Medicine, and Psychiatry’s special issue on Ethnography and Social Medicine (Buchbinder at al., 2025), by Kim Sue (2025) in New York City’s Rikers Island gulag and Liza Buchbinder (2025) in the even larger carceral sprawl of Southern California; for exceptional U.S. psychiatrist/researcher critiques that pay close attention to the meaning of psychosis with their patients in therapy see (Braslow et al., 2021) and (Kalofonos, 2023), who also includes an interesting Fanonian sociogenic analysis of his patient's psychosis.

5 Personal communication from Blake Erickson who shadowed LA Department of Mental Health outreach violence de-escalation teams that counter-productively had law enforcement officer “accompaniment”. An accompanying officer abruptly sabotaged the outreach team’s attempt to gently de-escalate a floridly psychotic distressed patient called in by the patient’s concerned mother. The officer sabotaged the de-escalation by calling LA’s notoriously violent Highway Patrol SWAT extraction team. When Erickson queried him about his decision to escalate to violence he responded dismissively, “Crime over crazy”.

6 Caricaturally, Trump monetized class-based judicial sentencing disparities. He dispensed a historically unprecedented number of presidential pardons to wealthy white-collar criminals condemned in civil courts—most convicted of fleecing millions of dollars from the governments and the public. Particularly notorious is Juan Orlando Hernández, a formerly incarcerated president of Honduras, who orchestrated the smuggling of ~400 tons of cocaine into the USA (Greenberg, 2026; US Department of Justice, 2024). These wealthy criminals only had to send the Trump campaign large enough contributions from behind bars or during their civil court trials. Life became militarized for undocumented immigrants. He massified the number of Immigration and Customs Enforcement (ICE) agents, exempting them from independent judicial oversight. Since 2025, they have become a black-masked paramilitary strike force rudimentarily trained to brutalize, detain, deport, and shoot on sight undocumented immigrants through systemic racialized profiling. He built hundreds of new carceral concentration camps for immigrants, terrifying millions more to “voluntarily” self-deport. He ended coherent record keeping for public law enforcement and border control to prevent transparency. Trump’s authoritarian decrees threaten the survival of millions of nuclear and extended family members dependent on U.S. wage remittances across the world (see the vitriol and falsified public statistics in Trump White House, 2026; TRAC, 2026a, 2026b).

7 For documentation of the liberation theology revolutionary peasant movement and the U.S. funded and armed military dictatorships dominating El Salvador, see Bourgois’ (2023) 1981 photo-ethnographic account of a scorched-earth operation led by U.S. counterinsurgency officers that massacred civilian supporters of a concrete utopian alphabetization, medical literacy, and civil rights movement. For ethnographic documentation of survivors of massacres a generation later, see the pediatrician/researcher Barnert’s (2023) ethnographic account of creating a liberation medicine genetic database crucial to the success of the survivors of massacres movement that has been reuniting the “disappeared generation” of orphans from the 1980-1992 Salvadoran revolutionary civil war with surviving extended kin despite fascist military dictators and oligarchical authoritarian kleptocrats being elected presidents. For documentation of the oldest, largest mass movement of landless small farmers in Brazil, see Flynn’s (2025) ethnographic analysis of the crucial utopian mobilization components that contribute to the squatter movement’s success and endurance against repressive odds and political dogmatism.

8 See the publications of the Mental Health Assistants at: https://inmatementalhealthassistants.com/, https://store.bookbaby.com/book/the-solution-mental-health-assistants, and https://books.apple.com/us/author/adrian-berumen/id1537422294.
